# Successful treatment of disfiguring facial scar using surgery combined with superficial radiotherapy: a case report with literature review

**DOI:** 10.3389/fonc.2025.1586196

**Published:** 2025-08-21

**Authors:** Yong-xia Zong, Yinghua Song, Shuang Deng, Liang Zhang, Ze Li

**Affiliations:** ^1^ School of Medicine, Jianghan University, Wuhan, China; ^2^ Department of Dermatology, Wuhan No.1 Hospital, Wuhan, Hubei, China; ^3^ Institute of Burns, Tongren Hospital of Wuhan University and Wuhan Third Hospital, Wuhan, China

**Keywords:** keloids, superficial radiotherapy, disfiguring scar, surgery, reshape

## Abstract

**Introduction:**

Facial scars are generally disfiguring and can cause both physiological and psychological trauma. Currently, there is a lack of effective treatment options for facial scars. In recent years, local superficial radiation therapy has emerged as a clinically proven treatment to effectively prevent scar recurrence after surgery.

**Case presentation:**

Our patient was a 41-year-old male with a disfiguring facial scar caused by extensive burns. The patient underwent surgery for his facial scar; however, the scar recurred after surgery. Then, the patient underwent superficial radiotherapy, after which the patient’s facial scar flattened gradually, and his face was restored to normal. No significant adverse reactions were observed after 1 year of follow-up.

**Conclusion:**

The results indicate that superficial radiotherapy may promote the atrophy of hypertrophic scar tissue and can restore the normal appearance of the skin by reshaping the wound repair process.

## Introduction

Keloids are common disfiguring fibroproliferative skin disorders characterized by increased proliferation of fibroblasts and excessive deposition of extracellular matrix, and an effective treatment for keloids is currently lacking (1). Many studies have demonstrated that postoperative radiotherapy at the wound site effectively reduces scar recurrence. The superficial radiotherapy system (SRT-100; Sensus Healthcare, Boca Raton, FL, USA) is an X-ray emission device that is precisely controlled by a computer (2). Compared with traditional radiation therapy schemes, superficial radiotherapy has the advantages of precise dosage, shallow penetration depth, safety and effectiveness, and fewer adverse reactions (3). We successfully treated a burn patient with facial scar recurrence after surgery using SRT-100. The detailed treatment process is described in the following section.

## Case presentation

**Figure 1:**
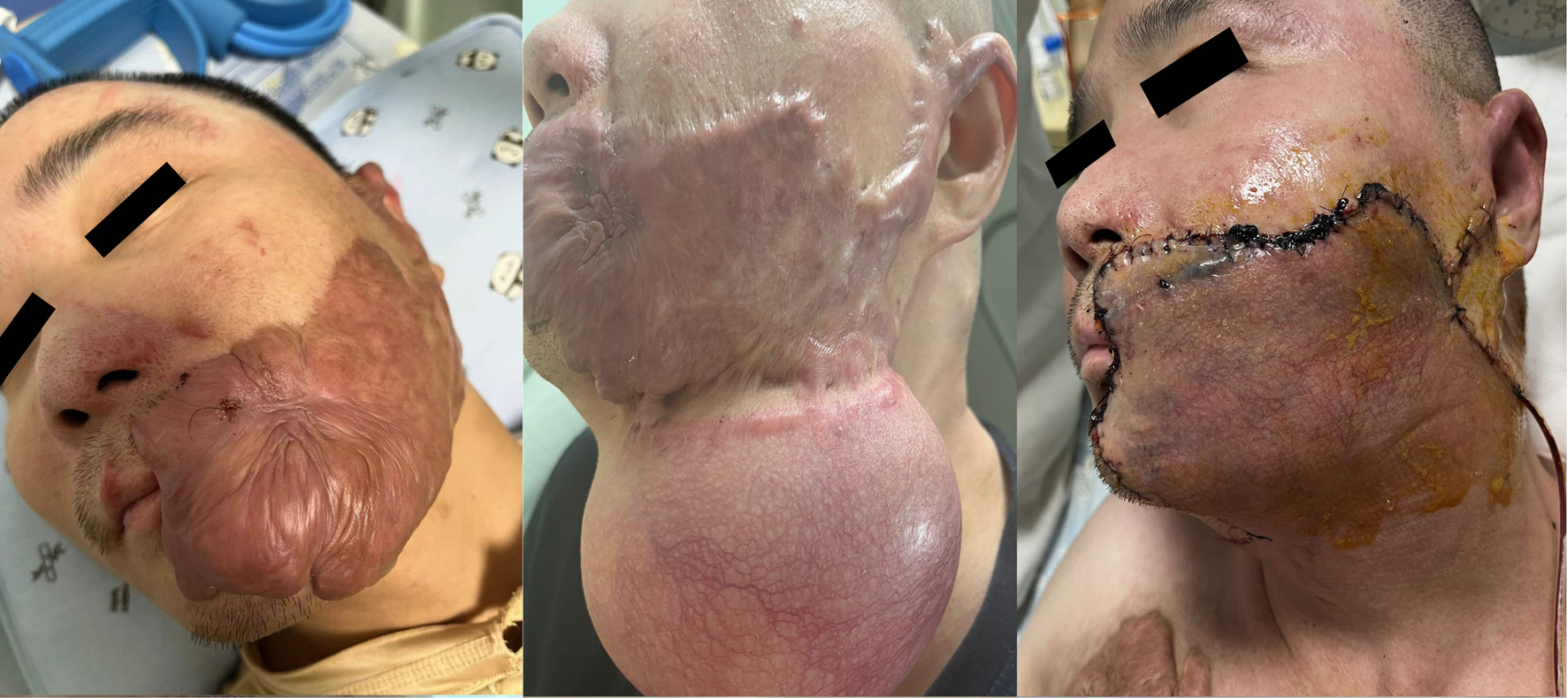
Patient with extensive facial scar tissue undergoing surgical resection and neck flap transfer for repair treatment. Left: A large scar covering most of the patient’s left cheek. Middle: Preparation of the neck skin by inserting a subcutaneous expander (200 ml). Right: Repair of residual wounds through neck flap transfer surgery after scar removal.

Our patient was a 41-year-old male who suffered extensive burns while working on a construction site in 2021 and received anti-infection and symptomatic treatment in the burn department of a local hospital. Large areas of hypertrophic scars were formed after wound healing. One of the larger scars was located on the surface of the skin near the left corner of the mouth, with locally hypertrophic scars covering most of the left cheek, disfiguring the patient’s left face (**Figure 1**, left). The patient underwent subcutaneous expander (200 ml) placement surgery for facial wound closure preparation after scar excision at a local burn hospital (**Figure 1**, middle). Physiological saline was injected into the expander to dilate the skin, with an injection frequency of once a week and approximately 5 minutes each time. The total amount of physiological saline injected when the expander was removed was 460 ml. Then, the patient underwent left cheek scar excision surgery, and the defect wound after scar excision was repaired using the expanded neck flap (**Figure 1**, right).

**Figure 2:**
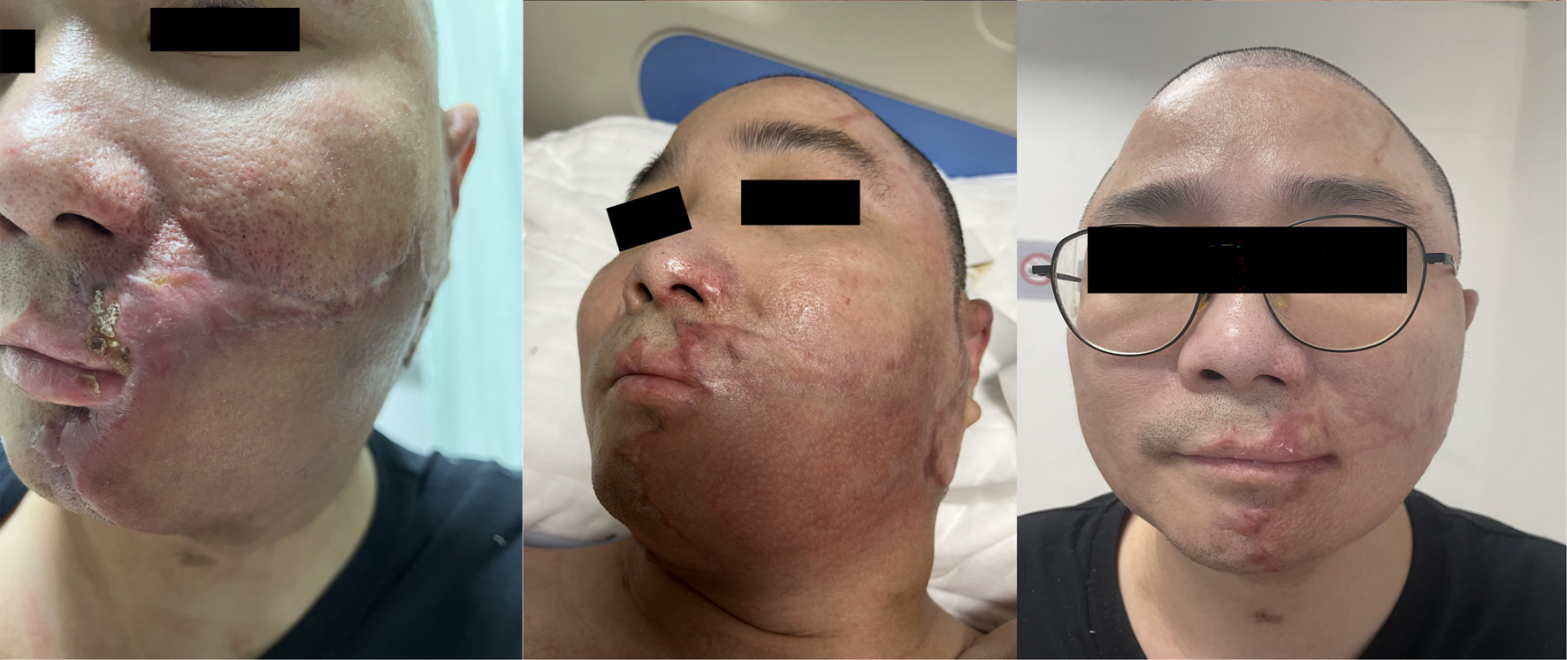
Superficial radiotherapy was performed after the recurrence of scar hyperplasia after surgery. Left: Proliferative scars recurred around the sutured wound margin of the transferred skin flap. Middle: At 4-month follow-up after superficial radiotherapy, hypertrophic scars flattened, and the appearance of the transferred skin flap was reshaped. Right: At 8-month follow-up after superficial radiotherapy, the patient’s left cheek returned to normal appearance.

Two days after surgery, the patient received superficial radiotherapy to the wound (SRT-100) once a day for 3 consecutive days, with a voltage of 50 kV and a single irradiation dose of 5 Gy. Twenty-two days after surgery, the patient’s wound healed, with hypertrophic scars appearing at the junction of the flap and normal skin. Furthermore, the healed flap was significantly higher than the surrounding normal skin (**Figure 2**, left). The patient completed the fourth round of superficial radiotherapy under the same voltage and radiation dose, with a total dose of 20 Gy. At 4-month follow-up after superficial radiotherapy, the patient’s facial hypertrophic scars significantly flattened, and the transferred skin flap survived well. The appearance was consistent with the surrounding normal skin surface (**Figure 2**, middle). At 8-month follow-up after superficial radiotherapy, the patient’s left cheek returned to its normal appearance (**Figure 2**, middle). The Vancouver scores before and after treatment were 14 and 5, respectively. During the treatment process and follow-up period, the patient did not experience any adverse reactions.

## Discussion

Keloids are benign, fibroproliferative dermal growths that occur in response to skin injury (4). Untreated scar tissue will gradually expand and extend toward the surrounding area, and the scar will gradually thicken over time, presenting a crab-foot-like appearance. Keloids are commonly refractory to many of the current treatments, and although many types of therapeutic methods have been suggested for treating this condition, there is currently no universally accepted method (5). If a keloid is simply surgically removed without other treatments, the scar is likely to recur, and its extent will be much larger than the surgical incision site. In severe cases, scar recurrence can affect the normal appearance of the patient’s skin or the function of nearby important joints. Surgical excision combined with adjuvant therapies such as post-operative radiotherapy is currently one of the recognized effective treatment options worldwide (6). For many years, the purpose of postoperative radiation therapy has been to reduce the recurrence rate of keloids. Common radiation therapy methods include X-ray, electron beam, brachytherapy, and heavy ion radiotherapy (7). The effect of various treatment options on the recurrence rate of scars varies, and the potential of radiotherapy as a treatment modality to induce malignancy has been of great concern. In the X-ray era before 1990, X-ray therapy was reported to have a zero carcinogenic rate, which provided supporting evidence for its clinical application (8).

The superficial radiotherapy system (SRT-100) is a recently emerging X-ray radiotherapy system, in which a computer is used to accurately calculate and deliver the doses so that the radiation can be concentrated on the superficial part of the skin while minimizing damage to normal cells. Therefore, superficial radiotherapy is suitable for areas where traditional radiotherapy is not suitable for application, such as areas that are too close to glands and certain important organs in the human body with poor radiation tolerance, such as the eyes. Because the rays of SRT-100 are precisely controlled, harmful and useless rays that scatter can be reduced to a minimum. SRT-100 can even be used to treat infantile hemangioma (3). The pathogenesis of keloid formation is not very clear, but it is mainly due to the abnormal proliferation of fibroblasts and excessive deposition of extracellular matrix. The basic principle of radiation therapy is to inhibit the proliferation and activation of fibroblasts, promote apoptosis of neovascular endothelial cells, and reduce excessive proliferation of scar tissue. The most concerning aspect of surgical excision combined with superficial radiotherapy for scar tissue is the issue of scar recurrence, and the recurrence rate corresponding to different radiation doses and fractions also varies (9).

Our patient received superficial radiotherapy within 48 hours after surgery, but 15 days after surgery, scar hyperplasia still appeared at the junction of the surgical wound and the skin flap, indicating that fibroblasts in the skin lesion were still actively proliferating. However, the hypertrophic scar tissue gradually shrank after superficial radiotherapy with sufficient treatment fractions and radiation doses. This result indicates that superficial radiation therapy can not only prevent the excessive proliferation of fibroblasts but also directly act on the surface of the scar tissue, promoting the atrophy and flattening of the already formed abnormal hypertrophic scar tissue. Based on this, for patients with keloid disease who cannot tolerate surgery in certain areas or patients who cannot undergo surgical treatment, direct superficial radiation therapy on scar lesions can promote the atrophy and flattening of scar tissue. In addition, superficial radiotherapy has a clear therapeutic effect and mild adverse reactions, and it is suitable for a wide range of people, making it one of the alternative non-invasive treatments for patients with keloids.

In our patient, after the wound on the left cheek was repaired with a neck flap, the surviving flap was significantly higher than the surrounding normal skin, and the appearance of the surviving flap was significantly different from that of the surrounding normal skin. At 6 months after superficial radiotherapy, the surviving skin flap was perfectly integrated with the surrounding normal skin, and its appearance was close to that of the surrounding normal skin. This result indicates that superficial radiotherapy has a reshaping effect on the skin flap; therefore, superficial radiotherapy may enable flaps from different sources to organically combine with the surrounding normal skin. The specific principle still requires further research and confirmation. Meanwhile, superficial radiotherapy still has adverse reactions, mainly including local pigmentation, radiation dermatitis, and delayed wound healing. Evaluating the skin sensitivity of patients receiving radiotherapy and adjusting the radiation dose, as well as timely assessment and appropriate management of radiation dermatitis, can effectively reduce the occurrence of such adverse reactions or alleviate the degree of damage caused by them.

## Data Availability

The original contributions presented in the study are included in the article/Supplementary Material. Further inquiries can be directed to the corresponding authors.
